# Gene expression profiles in human gastric cancer: expression of maspin correlates with lymph node metastasis

**DOI:** 10.1038/sj.bjc.6602429

**Published:** 2005-03-15

**Authors:** M Terashima, C Maesawa, K Oyama, S Ohtani, Y Akiyama, S Ogasawara, A Takagane, K Saito, T Masuda, N Kanzaki, S Matsuyama, Y Hoshino, M Kogure, M Gotoh, M Shirane, K Mori

**Affiliations:** 1 Department of Surgery 1, Fukushima Medical University, Fukushima 960-1295, Japan; 2 Department of Pathology, Iwate Medical University, Morioka 020-8505, Japan; 3 Department of Surgery 1, Iwate Medical University, Morioka 020-8505, Japan; 4 Product Research Department, Chugai Pharmaceutical Co., Ltd., Kamakura 247-8530, Japan

**Keywords:** gastric cancer, gene expression profiles, oligonucleotide microarray, maspin

## Abstract

To seek for a candidate gene that would regulate tumour progression and metastasis in gastric cancer, we investigated gene expression profiles by using DNA microarray. Tumour tissue and adjacent normal tissue were obtained from 21 patients with gastric cancer and then examined for their gene expression profiles by the Gene Chip® Human U95Av2 array, which includes 12 000 human genes and EST sequences. A total of 25 genes were upregulated and two genes were downregulated by at least four-fold in the tumour tissue. In a further analysis according to lymph node metastasis, the expressed levels of maspin, as well as carcinoembryonic antigen and nonspecific crossreacting antigen were significantly higher in tumours with lymph node metastasis than in those without it. Maspin expression in 85 gastric cancer patients was further investigated by using immunohistochemistry. Maspin expression was not observed in normal gastric epithelia without intestinal metaplasia. In contrast, maspin was expressed in 74 of 85 tumour tissues. There was a significant correlation between the incidence of maspin-positive tumour staining and lymph node metastasis. These results suggest that maspin has a potential role for tumour metastasis in gastric cancer.

The incidence of gastric cancer has been gradually declining in developed countries; however, gastric cancer is still the most common cancer in Eastern Asia, Eastern Europe, and South America and is the second leading cause of cancer deaths in the world (Pisani *et al*, 1999). For the treatment of gastric cancer, several modalities are performed such as surgery, chemotherapy, radiotherapy, and immunotherapy. Among them, surgery is the most comprehensive and reliable procedure to cure the disease, especially when it is localised. Nevertheless, the operative results for advanced gastric cancer remain unsatisfactory even when extensive surgery had been performed (Roukos, 2000). To improve the therapeutic results for gastric cancer, the exploration of genes related to metastasis and elucidation of the mechanism of tumour progression are considered to be of great importance. For this purpose, numerous molecular biological studies have been carried out, and several genetic and epigenetic alterations have been clarified for carcinogenesis and progression of gastric cancer. Inactivation of tumour suppressor genes such as *p53, DCC, APC, and E-cadherin* and amplification of oncogenes, including K-ras, beta-catenin, c-erbB-2, K-sam, cyclin E, and c-MET, are frequently observed in gastric cancer (Yasui *et al*, 2001). In addition, aberrant DNA methylation of promoter region CpG islands has been reported as a means for the inactivation of tumour suppressor or tumour-related genes (Tamura *et al*, 2000; Tamura, 2002).

More recently, microarray technology has made it possible to comprehensively analyse gene expression profiles (Hasegawa *et al*, 2002; Hippo *et al*, 2002; Inoue *et al*, 2002). By using this technique, the expression levels of thousands of genes can be analysed in a single experiment. This technology is a powerful tool for analysing gene expression profiles related to the development and progression of specific diseases. Although there have been significant improvements in the analysis of genetic alterations for gastric cancer, there is still insufficient information for understanding a common pathway for the development and progression of gastric cancer. Gastric cancer has diverse clinical properties such as histological type, metastatic status, race, and gender. Thus, further exploration to search for genetic alterations in gastric cancer is required.

In the present study, we performed oligonucleotide microarray analysis representing more than 12 000 unique genes to characterize the global gene expression profiles of gastric cancer tissues and sought to identify specific gene sets that correlate with lymph node metastasis.

From the microarray analysis, maspin was selected as one of the genes upregulated in gastric cancer. We had previously investigated the expression of maspin in gastric cancer and had reported that maspin expression was observed in 80% of gastric cancer tissues and in all gastric normal epithelium with intestinal metaplasia, but not in normal epithelium without intestinal metaplasia, and that expression of maspin was regulated by methylation at the CpG islands of the promoter region (Akiyama *et al*, 2003). Son *et al* (2002) also reported that 90% of gastric cancer tissue showed positive expression of maspin; however, the role of maspin in the progression of gastric cancer has not yet been elucidated. In the present study, we further investigated the correlation of maspin expression and clinicopathologic features with special reference to lymph node metastasis in gastric cancer.

## MATERIALS AND METHODS

### Tissue samples for DNA microarray

For DNA microarray, 21 pairs of gastric cancer tissue and corresponding gastric normal epithelium were obtained from a total of 21 patients who underwent gastrectomy at Iwate Medical University. Patient characteristics were determined according to the Japanese classification of Gastric Carcinoma (2nd English edition) (Japanese Gastric Cancer Association, 1998) and are listed in Table 1. Permission of the Institutional Review Board (IRB) was obtained (#H12-32, March 14, 2001), and written consent was obtained from all patients prior to surgery. Primary gastric cancer tissue and adjacent normal mucosa were carefully dissected immediately after operation from surgically resected samples and stored at −80°C until used for analysis. Tissue sections for histopathologic examination were made from the samples obtained to evaluate the presence of cancer cells in the tumour samples and the presence of intestinal metaplasia in the gastric normal epithelium. Of 21 normal samples, 15 contained intestinal metaplasia and were excluded from the normal controls. As a result, six samples were provided as normal controls.

### DNA microarray

A total of 21 gastric cancer tissues and six gastric normal epithelia were lysed and total RNA was extracted by using the Sepasol-RNA I (WAKO, Osaka, Japan) according to the manufacturer’s instructions. The extracted total RNA was purified with an RNeasy column (Qiagen, Austin, TX, USA). Affymetrix (Santa Clara, CA, USA) microarray analysis was performed according to the manufacturer’s instructions. Total RNA (5 *μ*g) was reverse transcribed to cDNA by using the T7-(dT) 24 primer. Biotin-labelled cRNA was synthesised from cDNA by using the MEGA script *In Vitro* Transcription Kit (Ambion, Austin, TX, USA). cRNA was fragmented to an average size of 50–100 nucleotides by incubation at 95°C for 35 min in 40 mM Tris-acetate (pH 8.1) containing 100 mM potassium acetate and 30 mM magnesium acetate, and then hybridisation to human GeneChip® (Human Genome Arrays U95Av2, Affymetrix) containing approximately 12 000 human genes. This commercially available array has been designed and used for quantitative and highly parallel measurements of gene expression (Lipshutz *et al*, 1999). The hybridised oligonucleotide microarrays were scanned with a confocal scanner (Affymetrix). The scanned data obtained from each microarray were normalised by the All Probe Set Normalization method, which adjusted the trimmed mean signal of the experiment to a global intensity of 300 units, to correct for small differences in the amounts of each cRNA probe applied to the microarray and were processed for average difference values by using Affymetrix software (LIMS 5.0). Fold level changes of gene expression in gastric carcinoma were calculated by comparison with the pooled data of six independent normal tissues.

### Real-time quantitative reverse transcription–polymerase chain reaction (RT–PCR) (RQ-PCR)

For RQ-PCR assay, the primers and fluorogenic probe were designed with Primer Express software (ABI): maspin F (nucleotides 646–665; 5′-CGA CCA GAC CAA AAT CCT TG-3′), maspin R (nucleotides 778–796; 5′-GAA CGT GGC CTC CAT GTT C-3′), probe (nucleotides 745–772; 5′-FAM-CAA CAA GAC AGA CAC CAA ACC AGT GCA G-TAMURA-3′). For RQ-PCR assay, an ABI PRISM7000 sequence Detector (ABI) was used. The reaction mix contained 50 ng of cDNA, 200 nmol l^−1^ of each primer, 5 *μ*mol l^−1^ of probe, and 25 *μ*l of TaqMan Universal PCR Master Mix (ABI), in a final volume of 50 *μ*l. The cDNA was subjected to 50 cycles of a two-step PCR consisting of a 15-s denaturation step at 95°C and a 1-min combined annealing/extension step at 60°C. Plasmids were diluted in a precise series, ranging from 5 pg to 0.005 fg (2 × 10^6^ to 2 copies). For normalisation of each target in the samples, the copy number of glyceraldehyde-3 phosphate dehydrogenase (GAPDH) was used as an internal control. Maspin and GAPDH expression levels (copy number) were calculated from standard curves using each plasmid. The normalised values of maspin mRNA were expressed as the ratio of maspin copy number per copy number of GAPDH.

### Immunohistochemistry

Tumour specimens and lymph nodes from 85 patients were fixed with 10% buffered formalin and embedded in paraffin. Patient characteristics are shown in Table 2. Tissue blocks were then sliced into 4-*μ*m sections and mounted on glass slides. The sections were pretreated in 10 mM citrate buffer (pH 6) by a microwave-based antigen-retrieval method for 15 min and then incubated overnight with monoclonal anti-human maspin antibody (Pharmingen International, San Diego, CA, USA) at a dilution of 1 : 50. The Histofine SAB-PO kit (Nichirei, Tokyo, Japan) was used to visualise the antibody binding. Stained slides were reviewed and graded by two pathologists by following a blind protocol (observers had no information on clinicopathologic data). The incidence of maspin-positive cells was graded as: 0, negative; 1+, <20%; 2+, 20–80%; 3+, >80%. Similarly, the relative staining intensities of tumour cells were graded as: 0, negative; 1+, weak; 2+, moderate; 3+, strong.

### Statistical analysis

For microarray analysis, all values were analysed by using the Mann–Whitney *U*-test or one-way analysis of variance (ANOVA) following Dunnett’s *t*-test. A *P*-values lower than 0.01 were considered to be statistically significant. For histochemistry, Fischer’s exact probability test and the *χ*^2^ test were used for testing the correlation between maspin protein expression and clinicopathologic factors. Statistical differences were evaluated between two groups with the Mann–Whitney test, and for three or more groups with the Kruskal–Wallis test. A *P*-value of less than 0.05 was considered to be statistically significant.

## RESULTS

### Commonly upregulated genes in gastric cancer by DNA microarray

We first applied a hierarchical clustering algorithm separately to the samples and genes by using the measure of similarity and average linkage clustering (Eisen *et al*, 1998) and found that tumour and normal tissues were divided into different groups (data not shown). We next searched for genes that are commonly upregulated or downregulated in gastric cancer. Fold change (FC) values of gene expression in the tumour tissue were calculated by comparison with the pool data of six independent normal tissues. We set the cutoff level of mean FC (MFC) at more than 4 or less than 0.25, and applied Mann–Whitney’s *U*-test to identify genes that were differentially expressed between cancer and normal tissue. As a result, we found that 25 genes were upregulated and two genes were downregulated in cancer tissue (*P*<0.01) (Table 2).

### Genes associated with lymph node metastasis

To identify genes associated with lymph node metastasis, we further compared gene expression profiles among six normal tissues, 16 cancers with lymph node metastasis, and five cancers without lymph node metastasis with respect to 27 genes (Table 3). Three genes showed significantly higher expression in cancers with metastasis than in those without metastasis (Table 4). If patients with hepatic or peritoneal metastasis were excluded, only maspin demonstrated significantly higher expression in cancers with lymph node metastasis than in those without it (*P*=0.0066).

We further investigated the expression of maspin by using RQ-PCR and immunohistochemistry.

### RQ-PCR for maspin mRNA

**Figure 1:**
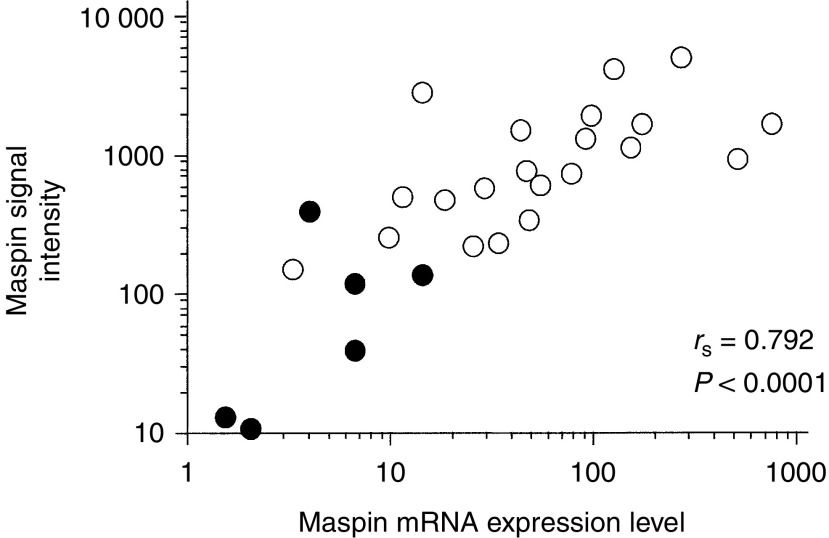
Correlation between relative signal intensities determined by DNA microarray and mRNA expression levels determined by RQ-PCR. Closed circles represent normal gastric epithelium (*n*=6), and open circles represent gastric cancer tissue (*n*=21).

To verify the reliability of DNA microarray analysis, we investigated the maspin mRNA expression level by RQ-PCR and compared the results with the signal intensity obtained from DNA microarray analysis for six samples from normal epithelium and 21 samples from gastric cancer. There was a significant correlation between the signal intensity of maspin and the maspin mRNA expression levels determined by RQ-PCR (Figure 1).

### Immunohistochemistry of maspin

**Figure 2:**
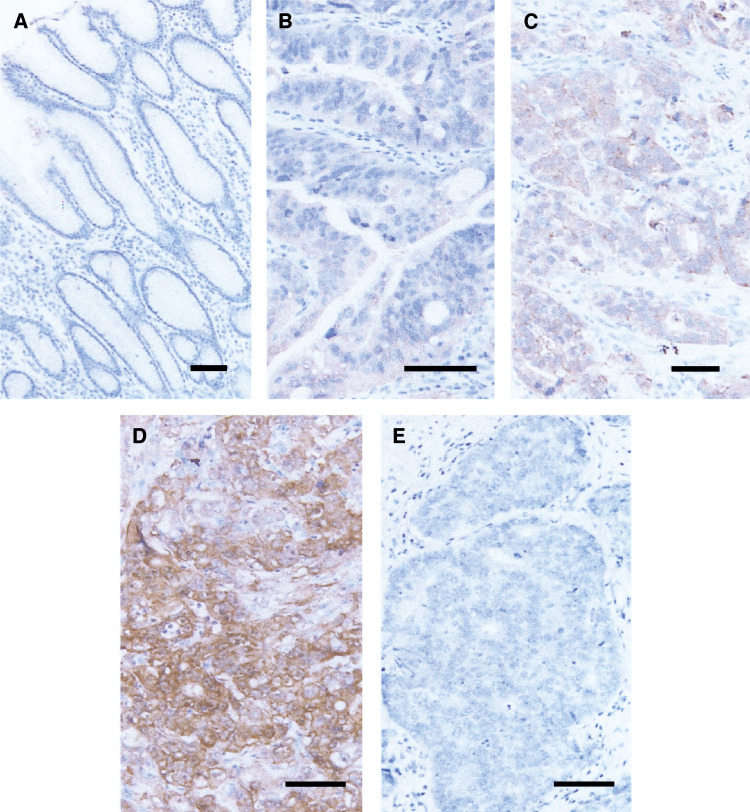
Immunohistochemistry for maspin protein. (**A**) High power view of gastric normal foverolar epithelium (scale bar=100 *μ*m). Immunoreactivity for maspin is negative. (**B**–**D**) High power view of maspin-positive gastric cancers (scale bar=100 *μ*m). Subcellular localisation of maspin protein is observed in cytoplasm and membrane (**B**–**D**). (**B**) (Case No. 24) and (**C**) (Case No. 32) are moderately differentiated tubular adenocarcinomas, and (**D**) (Case No. 14) is a poorly differentiated adenocarcinoma, solid type. (**E**) High power view of maspin-negative gastric cancers (Case No. 10; scale bar=100 *μ*m).

**Figure 3:**
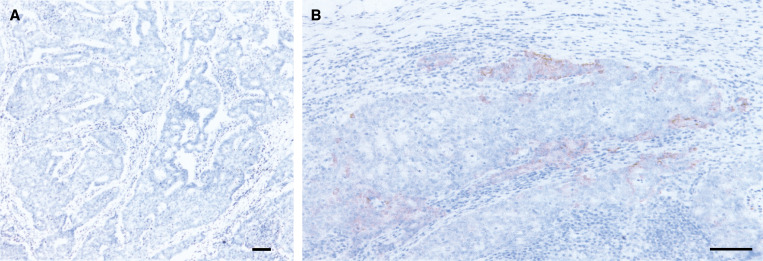
Immunohistochemistry for maspin protein in metastatic tumour cells of a lymph node (Case No. 10). (**A**) Low power view of a lymph node (Scale bar=100 *μ*m). Nests of metastatic tumour cells are observed in the lymph node periphery. (**B**) High power view of a metastatic lesion (scale bar=100 *μ*m). Focal and dense immunoreactivity is observed.

Immunohistochemistry was performed for a total of 85 patients including the 21 patients whose tissue samples were analysed by DNA microarray. Subcellular localisation of maspin protein was observed in both the cytoplasm and membrane (Figure 2B–D). Cells with nuclear localisation were extremely rare. A total of 78 cases displayed epithelium with intestinal metaplasia. Dense immunoreactivity was observed in the epithelium with intestinal metaplasia. Gastric normal epithelium without intestinal metaplasia showed no diffuse and dense immunoreactivity (Figure 2A). Maspin expression was observed in 74 of 85 samples (87%) from gastric cancer. Diffuse expression of maspin protein was observed in 43 of 85 samples (51%) and, the expression was relatively strong in 59 of 85 samples (69%). There was no significant correlation between maspin protein expression and clinicopathologic features such as depth of tumour invasion, hepatic or peritoneal metastasis, and histological type. When patients with peritoneal or hepatic metastasis were excluded, however, the incidence of maspin protein expression was significantly higher in tumours accompanied with lymph node metastasis (Table 5). We also examined immunoreactivities of metastatic tumour cells in 41 cases with lymph node metastasis. Five of the 41 cases with lymph node metastasis showed no positive immunoreactivity for maspin in the primary tumours. In four of these five cases, there was no positive immunoreactivity for maspin observed in the lymph node metastasis as well. However, one (Case No. 10) of the five cases exhibited positive staining for maspin in only the metastatic lesions of its lymph nodes (Figure 3). In all 36 cases showed positive staining in both primary and metastatic lesions.

## DISCUSSION

For the purpose of seeking commonly upregulated genes in specific tumour types, generally, a microdissection procedure is used to separate the cancer cells and parenchymal cells. However, gastric cancer tissue contains many parenchymal cells such as fibroblasts, smooth muscle cells, and tumour-infiltrating mononuclear cells, and there are numerous interactions between the cancer cells and parenchymal cells of such substances as angiogenesis inducing factor, growth regulatory cytokines, lectins, and MMPs (Arias, 2001). For this reason, it is very important to investigate the gene expression profiles not only in gastric cancer cells but also in the accompanying parenchymal cells. Thus, we evaluated the gene expression profiles in gastric cancer with stromal cells as a whole.

It has been reported that genes related to cell cycle regulation, growth factor, DNA synthesis, transcription factor, ubiquitin–proteasome pathway, matrix metalloproteinases, and angiogenesis-inducing factor are commonly upregulated in gastric cancer, as evidenced by using microarray technology (Hasegawa *et al*, 2002; Hippo *et al*, 2002; Inoue *et al*, 2002). In the present study, we also demonstrated the upregulation of previously reported genes such as Topo II, cdc28 protein kinase2, TIMP, collagen alpha-2 type I, Diubiquitin, and cyclin-selective ubiquitin carrier protein. We also observed the upregulation of genes for previously well-known tumour antigens overexpressed in gastric cancer carcinoembryonic antigen (CEA) and nonspecific crossreacting antigens (NCAs). Furthermore, we detected the upregulation of several interesting genes that have not been reported as being expressed in gastric cancer, such as CPE-R, Rvp.1, sarcolectin, KOC, Notch3, and maspin.

Among the upregulated genes, CEA, NCA, and maspin were selected as genes related to lymph node metastasis in the present study. CEA is also well known as a tumour marker in gastric cancer. It has been reported that serum levels and tissue expression of this protein correlate with evidence of tumour progression, such as lymph node metastasis, in keeping with our results (Kojima *et al*, 1984; Nakane *et al*, 1994; Kim *et al*, 2000). Nonspecific crossreacting antigen is a member of the CEA family and the specific upregulation of its mRNA or protein in cancer tissue compared with that in normal mucosa in gastric cancer, especially in well-differentiated adenocarcinoma, has been reported (Kodera *et al*, 1993; Kinugasa *et al*, 1998). There is no report, however, indicating a positive correlation between the expression of NCA and evidence of tumour progression such as lymph node metastasis. Although this is the first report describing the role of NCA in lymph node metastasis, because the number of patients was limited, further investigations to elucidate the role of NCA in tumour progression should be carried out.

Maspin is a serine protease inhibitor belonging to the serpin family. The *maspin* gene was originally identified in normal human breast epithelium as encoding a 42 kDa cytoplasmic protein, and is known to have tumour suppressive activity attributable to the inhibition of breast cancer cell motility, invasion, and metastasis (Sheng *et al*, 1994; Zou *et al*, 1994; Seftor *et al*, 1998). Similarly, the tumour suppressive activity of maspin has been demonstrated for prostate cancer (Umekita *et al*, 1997), oral carcinoma (Xia *et al*, 2000), and colon cancer (Song *et al*, 2002). The prognostic significance of maspin has also been demonstrated in breast cancer (Seftor *et al*, 1998) and squamous cell carcinoma of the oral cavity (Xia *et al*, 2000). On the contrary, overexpression of maspin was observed in pancreatic (Maass *et al*, 2001) and ovarian cancer (Sood *et al*, 2002). In the pancreas and ovary, maspin expression was not detected in normal epithelium; however, it was detected in cancer tissues, especially those with aggressive properties. Recently, Heighway *et al* (2002) also reported that maspin, as well as S100A2, was selected as an overrepresented gene in non-small-cell lung cancer by using cDNA microarray. Little has been reported on the role of maspin in gastric cancer. Son *et al* (2002) first investigated maspin expression by using immunohistochemistry and RT–PCR. They reported that 90% of carcinoma tissues showed immunoreactivity for maspin, and mRNA expression of maspin was significantly higher in gastric adenocarcinoma than in the gastric normal epithelium. They also fund that gastric epithelial cells with intestinal metaplasia showed positive staining for maspin. We had also previously reported that maspin expression was observed in 80% of gastric cancers, in all normal epithelia with intestinal metaplasia, but not in normal epithelium without intestinal metaplasia (Akiyama *et al*, 2003). In the present study, overexpression of maspin in carcinoma tissue compared with that of normal epithelium was also observed by using oligonucleotide microarray analysis, supporting the results of previous reports. The results of microarray analysis were verified by RQ-PCR. There was a strong correlation between the signal intensity of maspin determined by DNA microarray and the maspin mRNA levels determined by RQ-PCR, suggesting the high reliability of oligonucleotide microarray. The role of maspin in tumour development and progression in gastric cancer was further investigated by immunohistochemistry. As previously reported, maspin immunoreactivity was obtained in 87% of carcinoma tissues and in most of normal epithelium with intestinal metaplasia; however, none of the normal epithelium samples without intestinal metaplasia showed positive immunoreactivity to maspin protein. In addition, there was a significant correlation between the immunoreactivity of maspin and the quantity of maspin mRNA expression determined by oligonucleotide microarray and RQ-PCR (data not shown).

Concerning the correlation between maspin expression and clinicopathologic features, we obtained significantly higher expression of maspin in tumours with lymph node metastasis than in those without metastasis. Although overexpression of maspin is present in the pancreas (Maass *et al*, 2001), ovary (Xia *et al*, 2000), gastric (Son *et al*, 2002) and non-small-cell lung cancer (Heighway *et al*, 2002), there have been no reports describing a positive correlation of the maspin mRNA expression and lymph node metastasis. Thus, this is the first report to demonstrate a correlation between maspin expression and lymph node metastasis. The role of maspin in lymph node metastasis was further verified by using immunohistochemistry; the positive correlation was observed between maspin expression and lymph node metastasis in a subgroup of patients without distant metastasis. This result indicates a more direct evidence for the role of maspin in lymph node metastasis, because other metastatic pathways such as haematological metastasis and peritoneal metastasis had been excluded in this subgroup. In addition, in some patients, maspin was upregulated only in the metastatic foci in lymph nodes, while maspin expression was negative in the primary region. Thus, there is a possibility that expression of maspin is regulated by an epigenetic event that is differently represented in different organs. Recently, Kim *et al* (2004) reported that the presence of cytoplasmic maspin was correlated with lower tumour stage and less lymph node involvement in lung cancers other than squamous cell carcinoma. The contradictory results may derive from different regulation mechanisms in different organs.

The regulation mechanism for maspin function is not fully elucidated. The loss of maspin gene expression with increasing malignancy is reportedly regulated at the transcriptional level in breast cancer (Domann *et al*, 2000). Recent studies have reported on the roles of cytosine methylation and chromatin condensation in the downregulation of maspin expression during neoplastic progression (Maass *et al*, 2002). We had previously reported that the maspin gene promoter region of all normal epithelia without intestinal metaplasia was hypermethylated on both alleles, whereas those regions with intestinal metaplasia frequently represented the haploid type of hypomethylation status, and demethylation frequently occurred and extended to both alleles in gastric cancer. This suggests that demethylation at the maspin gene promoter disrupts the cell-type-specific gene repression in both normal epithelium and gastric cancer (Akiyama *et al*, 2003).

In conclusion, DNA microarray technology is thought to be a useful tool for identifying specific genes correlated with tumour progression in gastric cancer. Maspin, which was selected as a lymph node metastasis-related gene by using DNA microarray analysis, is upregulated in most gastric cancer tissues, and this gene is supposed to have a potential role in lymph node metastasis.

## Figures and Tables

**Table 1:** Patient characteristics for tissue analysis by DNA microarray^a^

*Gender*		*Lymph node metastasis* b	
Male	11	N0	5
Female	10	N1	11
			
*Age (years)*		N2	4
38∼85 (Avg: 61)		N3	1
			
*Peritoneal metastasis* c		*Histologic type* d	
P0	14	tub1	4
P1	7	tub2	3
		por1	7
			
*Cytology* e		por2	5
CY0	19	sig	1
CY1	2	ads	1
			
*Hepatic metastasis* f			
H0	20		
H1	1	*Stage*	
		Ia	2
*Depth of invasion* g		Ib	3
T1	2	II	2
T2	7	IIIa	3
T3	11	IIIb	2
T4	1	IV	9

a Patient characteristics were determined according to the Japanese Classification of Gastric Carcinoma (2nd edition).

b N0, none; N1, metastasis to Group 1 lymph nodes; N2, metastasis to Group 2 lymph nodes; N3, metastasis to Group 3 lymph nodes.

c P0, absent; P1, present.

d tub1, well-differentiated tubular adenocarcinoma; tub2, moderately differentiated tubular adenocarcinoma; por1, poorly differentiated adenocarcinoma (solid type); por2, poorly differentiated adenocarcinoma (non-solid type); sig, signet-ring cell carcinoma; ads, adenosquamous carcinoma.

e Cytology in peritoneal washings; CY0, negative; CY1, positive.

f H0, absent; H1, present.

g T1, within mucosa or submucosa; T2, until muscularis propria or subserosa; T3, penetration of serosa; T4, invasion of adjacent structures.

**Table 2:** Patient characteristics for tissue analysis by immunohistochemistry^a^

*Gender*		*Lymph node metastasis* b	
Male	11	N0	41
Female	29	N1	24
			
*Age (years)*		N2	15
18–86 (Avg: 68)		N3	5
			
*Peritoneal metastasis* c		*Histologic type* d	
P0			
P1	16	pap	3
		tub1	25
		tub2	19
			
*Cytology* e			
CY0			
CY1	9	por1	17
		por2	13
		sig	4
			
*Hepatic metastasis* f			
H0			
H1	7	muc	3
		ads	1
			
*Depth of invasion* g			
T1	30		
T2	27	Stage	30
T3	24	Ia	11
T4	4	Ib	10
		II	4
		IIIa	5
		IIIb	25
		IV	

a Patient characteristics were determined according to the Japanese Classification of Gastric Carcinoma (2nd edition).

b N0, none; N1, metastasis to Group 1 lymph nodes; N2, metastasis to Group 2 lymph nodes; N3, metastasis to Group 3 lymph nodes.

c P0, absent; P1, present.

d pap, papillary adenocarcinoma; tub1, well-differentiated tubular adenocarcinoma; tub2, moderately differentiated tubular adenocarcinoma; por1, poorly differentiated adenocarcinoma (solid type); por2, poorly differentiated adenocarcinoma (nonsolid type); sig, signet-ring cell carcinoma; muc, mucinous adenocarcinoma; ads, adenosquamous carcinoma.

e Cytology in peritoneal washings; CY0, negative; CY1, positive.

f H0, absent; H1, present.

g T1, within mucosa or submucosa; T2, until muscularis propria or subserosa; T3, penetration of serosa; T4, invasion of adjacent structures.

**Table 3:** Genes commonly upregulated and downregulated in gastric cancers

**Accession ID^a^**	**Gene name**	**Normal**	**Tumour**	**MFC^b^**	***P* c**
*Upregulated genes*					
AB000712	CPE-receptor	221.50	2204.38	9.95	2.10E-06
M18728	Nonspecific crossreacting antigen	241.22	2275.94	9.44	5.40E-03
M29540	Carcinoembryonic antigen	262.13	2364.40	9.02	1.66E-03
AJ238246	Sarcolectin	82.90	726.15	8.76	2.82E-03
D78611	MEST	45.57	399.95	8.76	2.67E-03
U04313	Maspin	226.72	1693.48	7.47	9.47E-04
L17131	High mobility group protein (HMG-I(Y))	307.70	1933.66	6.28	1.16E-05
AL031983	dJ271M21.6 (Diubiquitin)	286.75	1741.25	6.07	2.39E-03
AA156240	Homo sapiens cDNA	280.58	1691.72	6.03	1.82E-04
J03464	Collagen alpha-2 type I	518.23	2991.88	5.77	1.67E-03
X54942	ckshs2	170.18	968.87	5.69	1.76E-05
U73379	Cyclin-selective ubiquitin carrier protein	283.25	1525.39	5.39	1.66E-06
AA976838	oq35c12.s1 Homo sapiens cDNA	155.18	835.00	5.38	5.84E-03
J04088	DNA topoisomerase II (Top2)	208.87	1092.85	5.23	9.23E-03
D80008	KIAA0186	73.47	364.53	4.96	1.28E-03
AA203476	zx55e01.r1 Homo sapiens cDNA	131.52	646.47	4.92	2.77E-07
U55206	Gamma-glutamyl hydrolase (hGH)	74.68	327.58	4.39	6.20E-04
J04152	Gastrointestinal tumour-associated	483.73	2028.00	4.19	1.51E-05
	Antigen GA733-1 protein				
U97188	Putative RNA-binding protein KOC	58.85	246.36	4.19	2.35E-04
D11139	Tissue inhibitor of	1047.67	4334.40	4.14	5.03E-03
	metalloproteinases				
U28386	Nuclear localisation sequence	122.48	505.54	4.13	1.65E-06
	receptor hSRP1 alpha				
U97669	Notch3	123.62	510.09	4.13	4.11E-04
AB000714	hRVP1	194.80	788.65	4.05	4.22E-03
					
*Downregulated genes*					
AB020629	KIAA0822	204.94	48.96	0.24	3.00E-4
AL050159	DKFZp586A0522	1940.37	434.71	0.224	1.89E-3

a Gene bank accession ID.

b MFC, median fold change; tumour *vs* normal mucosa (T/N) gene expression values.

c Calculated by Mann–Whitney’s *U*-test.

**Table 4:** Genes with expression altered between node-positive (*n*=16) and node-negative (*n*=5) tumours

		**MFC^b^**		
**Accession ID^a^**	**Gene name**	**Node-negative**	**Node-positive**	***P* c**
U04313	Maspin	2.79	11.82	0.0019
M18728	Nonspecific crossreacting antigen	2.48	10.90	0.0157
M29540	Carcinoembryonic antigen	3.43	10.20	0.0295

a Gene bank accession ID.

b MFC, mean fold change; tumour *vs* normal mucosa (T/N) gene expression values.

c Calculated by Mann–Whitney’s *U*-test.

**Table 5:** Correlation between lymph node metastasis and imunohistochemistry for maspin protein in 65 patients without distant metastasis

**Tumour**	**Number of patients**	**Incidence of maspin-positive cells**				
		**0**	**1**	**2**	**3**	
Node-negative	41	6	4	15	16	*P*=0.0133^a^
Node-positive	24	1	2	2	19	

a *χ*^2^ test.
